# Targeting OSMR in glioma stem cells

**DOI:** 10.18632/oncotarget.15066

**Published:** 2017-02-03

**Authors:** Sushmetha Mohan, Azad Bonni, Arezu Jahani-Asl

**Affiliations:** Department of Oncology, Integrated Program in Neuroscience, McGill University, Lady Davis Institute for Medical Research, Jewish General Hospital, Montreal, Quebec, Canada; Department of Neuroscience, Washington University School of Medicine, St. Louis, MO, USA

**Keywords:** OSMR, EGFRvIII, STAT3, tumor stem cells, glioblastoma, Neuroscience

The median survival rate for patients with glioblastoma tumors remains less than 18 months despite intense efforts and advances in surgery, radiation and chemo-radiotherapy [1]. There is, therefore, an urgent need for better treatment of these malignant tumors. Although glioblastoma tumors are composed primarily of astrocyte-derived cells, a population of stem-like cells within the tumor mass is thought to contribute to the growth of these tumors, their resistance to therapies, and tumor recurrence following surgery. These cells, termed brain tumor stem cells (BTSC) or glioma stem-like cells (GSC), behave as tumor initiating cells as a small population can give rise to secondary tumors when transplanted into immunodeficient mice [2]. Interestingly, the xenografted tumors in mice resemble primary tumors in patients, and exhibit similar histological and cytological features and genetic heterogeneity.

Approximately 50% of glioblastoma patients harbor amplification of the gene encoding epidermal growth factor receptor (EGFR), and more than half of these patients also carry the truncated active EGFR mutant EGFRvIII [3, 4]. EGFRvIII is constitutively active and functions as a ligand-independent receptor leading to sustained induction of oncogenic pathways including the activation of the transcription factor STAT3 [5]. Targeting EGFR and EGFRvIII in high-grade glioma remains a major focus of research in neuro-oncology. To date, however, tyrosine kinase inhibitors and EGFRvIII antibodies have not yielded successful results in glioblastoma patients.

In characterizing the basic biology of EGFRvIII-expressing BTSCs, we have identified the receptor for the cytokine Oncostatin M (OSMR) as a novel key regulator of BTSC proliferation and glioblastoma tumorigenesis [6–8]. RNA-seq analyses of human BTSCs reveals that OSMR is upregulated in an EGFRvIII- and STAT3-dependent manner. Importantly, OSMR mRNA is significantly upregulated in glioblastoma patients and correlates with poor prognosis. Multivariate analyses of human patient databases deposited at TCGA and REMBRANDT shows that OSMR is an important predictor of survival, even when controlling for other factors that impact patient survival including patient age, tumor grade, STAT3 expression, and IDH1 status. Tumor assays using human BTSC xenografts in immuno-deficient SCID mice reveals that knockdown of OSMR impairs their ability to form tumors *in vivo*. Further, mice transplanted with OSMR knockdown BTSCs survive significantly longer than mice transplanted with control BTSCs that express OSMR. Together, these findings highlight the significance of OSMR as a potential druggable target in glioblastoma therapy. Although designing OSMR-targeted antibodies and screens for drugs to inhibit OSMR represent key approaches to shut down OSMR-mediated signaling in glioblastoma tumors, a better understanding of OSMR function and its regulation in these tumors is required for better drug design.

**Figure 1:**
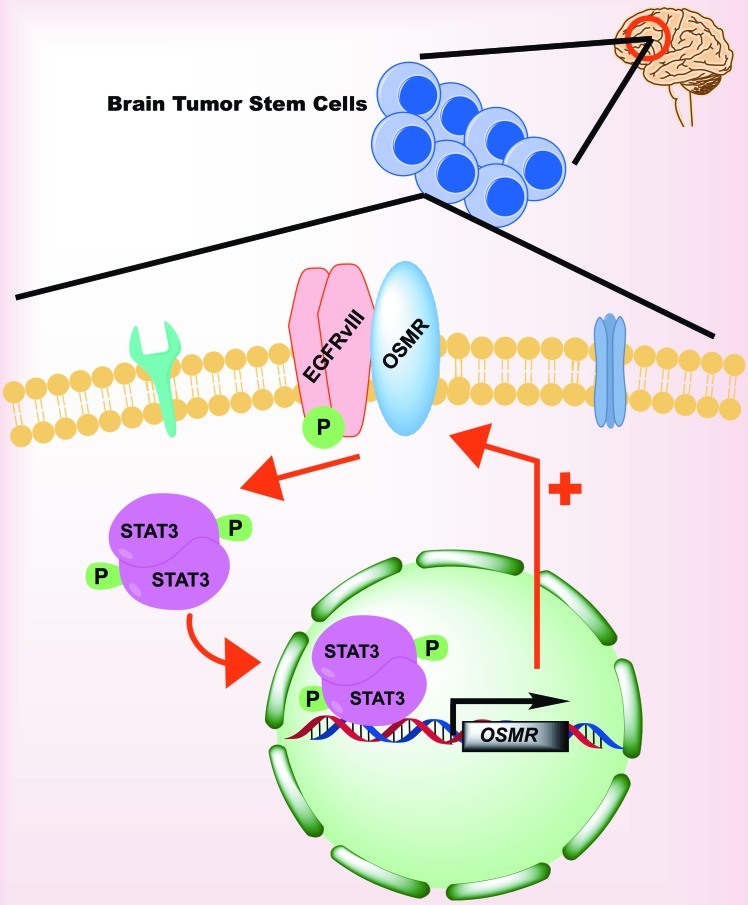
OSMR signaling in Glioma Stem Cells

Classical biochemical methods including co-immunoprecipitation, immunoblotting and immunohisto-chemistry, in parallel with contemporary imaging techniques such as proximity ligation assay and ligand response studies suggest that OSMR and EGFRvIII form a co-receptor complex on the cell membrane (Figure 1). Importantly, the ligand OSM regulates EGFR phosphorylation in wild type mouse astrocytes, and knockdown of OSMR in EGFRvIII-expressing astrocytes reduces the phosphorylation and total levels of EGFRvIII. Whether OSMR regulates the stability of EGFRvIII remains a subject for future studies. Further, proteins that regulate the EGFRvIII/OSMR co-receptor complex and downstream molecular pathways remain to be investigated. In view of the findings that OSMR impairs EGFRvIII phosphorylation, designing small peptides that interfere with the OSMR/EGFRvIII interaction provide an alternative attractive approach to suppress OSMR in glioblastoma cells.

ChIP-seq analyses of EGFRvIII-expressing mouse astrocytes singled out OSMR as a direct STAT3 target. ChIP-PCR analyses of EGFRvIII-expressing human BTSCs were performed to confirm that STAT3 directly binds the promoter of OSMR to upregulate its expression in different human BTSC lines (Figure 1). Knockdown of STAT3 in human BTSC and conditional knockout of STAT3 in mouse astrocytes reduces OSMR mRNA and protein expression levels in these cells. These data have established that OSMR is a direct target of STAT3. Therefore, OSMR mediates STAT3-induced oncogenic signaling as a direct transcriptional target, while at the same time OSMR functions upstream of STAT3 as a co-receptor with EGFRvIII to activate STAT3. This positive feed-forward mechanism appears to maintain EGFRvIII/STAT3 oncogenic signaling in BTSC.

Gene expression profiling of OSMR target genes and their intersection with STAT3- or EGFRvIII-target genes in mouse astrocytes demonstrates that while OSMR shares an overlapping network of 89 genes with STAT3 and 44 genes with EGFRvIII, OSMR regulates more than 300 unique targets that are not regulated by either of STAT3 or EGFRvIII. This suggests the hypothesis that OSMR may possess additional functions in oncogenesis beyond the regulation of the EGFRvIII/STAT3 oncogenic pathway. Investigation of OSMR’s unique gene signature as well as structure function analyses of OSMR should shed light on the full spectrum of OSMR activities in cancer and its value in glioblastoma therapy.
